# Structural Health Monitoring of Chemical Storage Tanks with Application of PZT Sensors

**DOI:** 10.3390/s23198252

**Published:** 2023-10-05

**Authors:** Michal Dziendzikowski, Paulina Kozera, Kamil Kowalczyk, Kamil Dydek, Milena Kurkowska, Zuzanna D. Krawczyk, Szczepan Gorbacz, Anna Boczkowska

**Affiliations:** 1 Airworthiness Division, Air Force Institute of Technology, ul. Ks. Boleslawa 6, 01-494 Warsaw, Poland; michal.dziendzikowski@itwl.pl (M.D.); kamil.kowalczyk@itwl.pl (K.K.); 2 Faculty of Materials Science and Engineering, Warsaw University of Technology, ul. Woloska 141, 02-507 Warsaw, Poland; kamil.dydek@pw.edu.pl (K.D.); milena.kurkowska@pw.edu.pl (M.K.); zuzanna.krawczyk2.stud@pw.edu.pl (Z.D.K.); anna.boczkowska@pw.edu.pl (A.B.); 3 Amargo Ltd., ul. Jasminowa 16, 05-850 Koprki, Poland; szczepan.gorbacz@amargo.pl

**Keywords:** structural health monitoring, chemical storage tank monitoring, hybrid material damage detection, PZT transducer applications

## Abstract

Chemical pressure storage tanks are containers designed to store fluids at high pressures, i.e., their internal pressure is higher than the atmospheric pressure. They can come in various shapes and sizes, and may be fabricated from a variety of materials. As aggressive chemical agents stored under elevated pressures can cause significant damage to both people and the environment, it is essential to develop systems for the early damage detection and the monitoring of structural integrity of such vessels. The development of early damage detection and condition monitoring systems could also help to reduce the maintenance costs associated with periodic inspections of the structure and unforeseen operational breaks due to unmonitored damage development. It could also reduce the related environmental burden. In this paper, we consider a hybrid material composed of glass-fiber-reinforced polymers (GFRPs) and a polyethylene (PE) layer that is suitable for pressurized chemical storage tank manufacturing. GFRPs are used for the outer layer of the tank structure and provides the dominant part of the construction stiffness, while the PE layer is used for protection against the stored chemical medium. The considered damage scenarios include simulated cracks and an erosion of the inner PE layer, as these can be early signs of structural damage leading to the leakage of hazardous liquids, which could compromise safety and, possibly, harm the environment. For damage detection, PZT sensors were selected due to their widely recognized applicability for the purpose of structural health monitoring. For sensor installation, it was assumed that only the outer GFRP layer was available as otherwise sensors could be affected by the stored chemical agent. The main focus of this paper is to verify whether elastic waves excited by PZT sensors, which are installed on the outer GFRP layer, can penetrate the GFRP and PE interface and can be used to detect damage occurring in the inner PE layer. The efficiency of different signal characteristics used for structure evaluation is compared for various frequencies and durations of the excitation signal as well as feasibility of PZT sensor application for passive acquisition of acoustic emission signals is verified.

## 1. Introduction

Chemical tanks are specialized containers designed to store hazardous substances. The most important considerations when designing them are as follows: operating temperature (mostly in the range between −20 and 40 °C), pressure and service environment, reliability, safety, design life, and costs. The vessel’s dimensions generally follow the design guidelines aimed at minimizing the risk of health and environmental hazards [1].

Chemical storage tanks can be categorized as either pressureless or low-pressure. However, even for pressureless tanks there is a possibility of achieving pressure levels of up to 3 bar during the last phase of unloading when the liquid is pushed out of the tanker by compressed air [2]. Trade organizations and engineering societies, such as the American Petroleum Institute (API), the American Institute of Chemical Engineers (AIChE), the American Society of Mechanical Engineers (ASME), and the National Fire Protection Association (NFPA) are responsible for developing engineering guidelines and standards for the construction, material selection, design, and safe management of storage tanks and accessories. A number of standards governing the design and manufacturing of chemical storage tanks have been released [1], in particular ISO 28300:2008 [3] and API STD 2000:2014 [4].

Materials for chemical tank manufacturing should feature high strength and stiffness. It is also desirable that the products be lightweight. Moreover, the chosen materials should provide excellent chemical and corrosion resistance. A stainless steel storage tank is well suited for storing high-temperature, high-volume, high-pressure acids and petroleum products. However, steel is a high-density material, leading to an increased tank weight and susceptibility to corrosion. An alternative are cost-efficient and low-weight polyethylene (PE) materials (linear, cross-linked, or high-density polyethylene), widely used in the manufacturing of chemical storage tanks. According to the ASTM D1998-21 standard [5], thanks to their chemical resistance, PE tanks can be operated in contact with aggressive reagents for extended periods of time. However, polyethylene may not be chemically resistant to some highly concentrated chemicals, additionally stored at elevated temperatures, such as concentrated sodium hypochlorite at above 40 °C. Replacing polyethylene with polyvinyl chloride (PVC) is not recommended as PVC is hard and stiff, which may cause cracks in the tank structure. In contrast, the use of chemically resistant polyvinylidene fluoride (PVDF) is an alternative, however, much more expensive solution. Ensuring chemical resistance when storing hazardous chemicals can also be effected through the use of double-layer tank walls. The internal thermoplastic liner provides chemical resistance, while the outer layer sustains the mechanical parameters. Additionally, if significant loads are expected to be exerted on a tank’s wall, e.g., due to the high capacitance of the tank or if a chemical medium needs to be stored under significantly high pressure, reinforcement of the structure with the use of another material featuring higher stiffness can be considered. For such applications, fiber-reinforced polymers (FRPs) are often the standard recommendation. Usually, composite pressure vessel and tanks can be operated within a temperature range of −40 °C to 120 °C [6,7]. The design, production, and testing requirements for FRP tanks and vessels, with or without a thermoplastic liner for storing fluids, whether non-pressurized or pressurized up to 10 bar, are outlined in three parts of the European standard EN 13121 [8].

Since their invention in the 1930s, glass fibers have consistently been the most popular type of fibers utilized in FRP composite applications. They are available in a variety of compositions, but, in general, two basic types can be distinguished. More than 90% of the glass fibers produced are general-purpose products. These types of glass fibers are referred to as E-glass, containing approximately 5% to 6% boron oxide by mass. Other glass fibers are premium special-purpose materials. The so-called ECR glass is known for its high corrosion resistance; this includes AR-resistant glass, which is alkali-resistant zirconium glass (with a zirconium oxide content of approximately 16–19%). It is used in construction in combination with cement-based materials. There are also other types of glass fibers, such as high-strength glass (types S, R, Te), low dielectric constant (D glass), and quartz/silica fibers that can withstand very high temperatures.

Owing to their highly attractive performance-to-price ratio, glass fiber products account for over 95% of fiber reinforcements used in the composite industry [9]. GFRPs exhibit high durability and strength, strong resistance to chemical and atmospheric agents, and low-density properties [10]. Their application can lead to significant weight reduction and cost savings compared to metallic structures, and consequently they are widely used in the automobile, aerospace, sports, and microelectronics industries. GFRPs are also used for chemical storage tank manufacturing.

Although GFRPs used for chemical storage tank fabrication are less expensive and more easily available than composites reinforced with other fibers, the cost of polyethylene tanks is lower, and their production is simpler. Additionally, glass fibers have an elongation limited to 3%, resulting in a low elasticity module of GFRP composites [6]. The production of a two-layer GFRP tank can be three times more expensive than the production of a polyethylene tank. However, when enhanced mechanical properties compared to PE-made tanks are required, strengthening the wall’s structure with GFRPs is a common choice for such applications. In addition, in cases involving highly aggressive reagents capable of penetrating the PE material, the use of a two-layer GFRP tank with a chemical-resistant layer made of PVDF material is more cost-effective than the use of a tank made exclusively from PVDF.

Polymers reinforced with glass fiber manufactured using the filament winding method are a popular choice for low-pressure tanks and pressure vessels. Filament winding is classified as a fiber in situ impregnation process because the fibers, after passing through separator combs, are impregnated by the polymer mixture in a resin bath on the production line. The wetted fibers are then wound onto a rotating mandrel. Once the appropriate thickness is achieved, the element undergoes curing and is removed from the mandrel. The filament winding process allows for an automation of the manufacturing of composites. It also facilitates the addition of high volumes of reinforcement compared to other methods. Moreover, it can be used to manufacture not only open elements, such as pipes, but also closed-end parts like pressure vessels [7,11].

Novel information-based approaches in the industry use data acquired through networks of sensors that monitor various parameters, which are important for control and optimization of industry processes, often employing artificial intelligence. In many areas, it is necessary to monitor the integrity of structures, which has led to the development of structural health monitoring (SHM) technologies [12,13,14,15,16]. Based on the modern approaches to machine learning (ML) and artificial intelligence, such as deep learning models, the data gathered by variety of sensors can be used to optimize the design, control and maintenance processes in the industry. Recent advancements in solving various engineering problems driven by the application of novel ML algorithms include the development of fiber optic sensors technology [17], signal denoising for electrical substations condition monitoring [18], development of guided-wave-based [19], vibration-based [20] or civil structures [21], structural health monitoring, performance improvement of oil and gas [22] or construction industries [23], as well as a number of other applications.

For chemical storage tanks, which can contain toxic or aggressive media under elevated temperatures and pressure, the application of an SHM system could significantly reduce the risk of sudden failures. Several market factors cause increased interest of manufacturers, users, and technical inspection authorities in the development of SHM systems for such structures. Currently, leakage detection systems are available for PE tanks, which generate alerts when structural integrity of the tank’s wall is compromised. Since chemical agents are usually hazardous to the environment, such events require immediate, unplanned maintenance actions, which can negatively affect production performance and increase overall maintenance costs. Additionally, for aggressive chemical substances stored at elevated pressures, the failure of a vessel’s integrity can jeopardize the safety of personnel or surrounding infrastructure. The development of systems designed for early damage detection could help prevent such incidents and optimize maintenance actions, including repairs, overhauls, or replacement.

Another potential advantage of introducing SHM systems to this market is related to service life extension and necessity of structure revisions. Chemical storage tanks typically follow the so-called safe-life approach in predefined periods of time, which often leads to their premature decommissioning, unnecessarily increasing the environmental footprint of the industry. They also require periodic inspections of both the external and internal structures, which, especially when conducted internally, require expensive and time-consuming preparation of vessels and can be hazardous to the operators. The development of reliable systems for loads and structural condition monitoring of chemical storage tanks could reduce the number of required inspections or even replace them. It could also provide the necessary data for implementing service life extension programmes, reducing costs and environmental impact of the storage of aggressive chemical agents.

**Figure 1:**
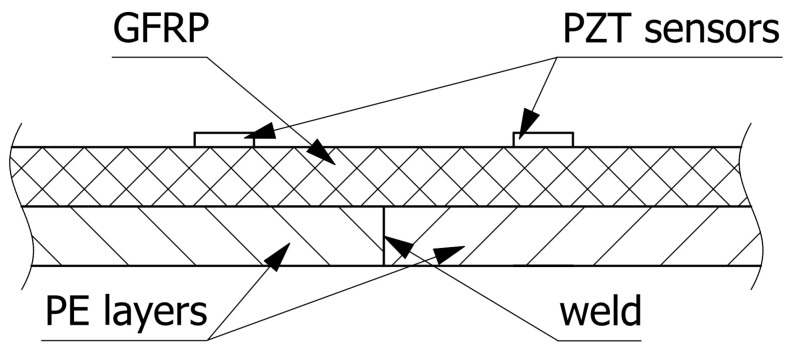
Chemical storage tank wall cross section.

Among various approaches to SHM [12,13,14,15,16], PZT ceramic sensors have proven to be a technology with universal application capabilities [24,25,26,27]. Elastic waves excited by PZT sensors can interact with any mechanical discontinuities caused by damage and can also penetrate various joints and interfaces. This enables them to be applied for the purpose of monitoring of complex structures. Figure 1 presents the cross section of a pressurized chemical storage tank wall. The outer GFRP layer of the tank provides the dominant part of the construction’s stiffness, while the inner layer, typically made of a PE material, is used to insulate chemical agents. The inner layer of the tank can be made using prefabricated PE sheets, which are then formed to the desired shape and thermally welded. The outer GFRP layer, especially in pressurized tanks, is typically wound around the inner PE layer; therefore, there are usually composite material joints. For an aggressive chemical medium, the sensors can be installed only on the outer layer as indicated in figure.

Guided waves excited by PZT sensors have proven to be effective in damage detection in composite materials, GFRPs in particular. Significant advancements in SHM systems using PZT transducers for composite structure monitoring have been achieved [26,27,28,29], particularly in impact damage detection and monitoring [30,31,32,33]. An identified matter of concern in the case of chemical storage tanks is the damage of the inner PE layer, especially at welded joints, such as cracks or erosion caused by exposure to a chemical medium. Therefore, the main focus of this work was to verify if elastic waves excited by PZT sensors installed on the outer GFRP layer are capable of penetrating the GFRP and PE interface, enabling their use for detection of damage in the weld. Additionally, we investigated the possibility of passive PZT sensor application to capture acoustic emission signals generated by the breakage of the internal GFRP structure.

The article is organized as follows. The next section describes the experiment and equipment used is provided. In Section 3, the methodology for damage detection both in active and passive operation modes of PZT sensors is delivered, followed by a discussion of the obtained results. Finally, the article is summarized.

## 2. Description of the Experiment

In this study, PZT sensors were selected as suitable for structural health monitoring of chemical storage tanks since guided waves excited by such transducers can travel significantly long distances considering the sensors’ dimensions and, theoretically, they can interact with all structure discontinuities caused by damage.

### 2.1. Preparation of Specimens

**Figure 2:**
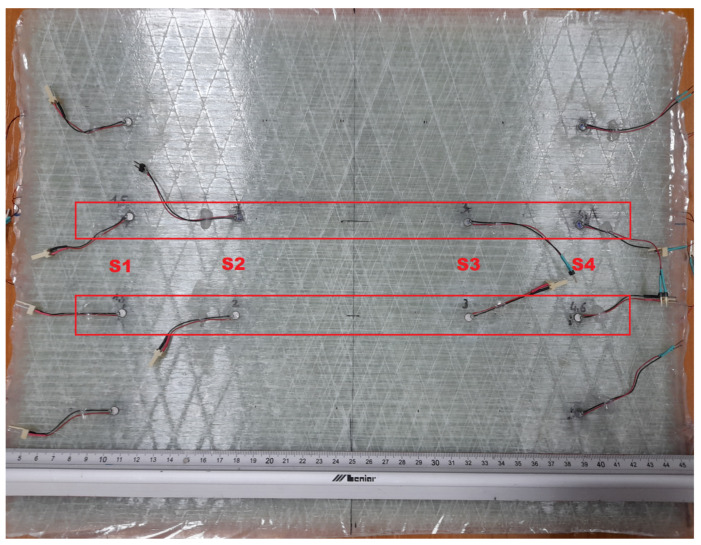
The test panel with indications of sensors used in the study.

In order to verify the efficiency of PZT sensors in damage detection of materials used in the manufacturing of chemical storage tanks, a flat hybrid panel similar to a tank structure was prepared. The panel was composed of a 5 mm thick GFRP layer providing structural stiffness, with an additional layer of PE material of the same thickness to be used for chemical protection. The cross section of the panel is presented in Figure 1. The GFRP material, consisting of twelve 400 mm × 300 mm plies, was manufactured by the hand lay-up technique. Derakane Momentum 470-300 resin, Butanox M-50, and cobalt naphthenate were used for composite production in the proportion 100/1/0.06 phr. The manufacturing process was conducted at ambient temperature and the composite was next post-cured at 80 °C for 2 h. A 5 mm thick layer of polyethylene was bonded to the composite layer using the Derakane Momentum 470-300 resin system. The polyethylene plate consisted of two elements thermally welded together. PZT sensors of the SMD05T04R111WL type by STEMINC Inc. [34] were installed on the composite layer of the specimen along the shorter edges of the panel, as presented in Figure 2.

Two independent PZT networks were installed on the panel for evaluating different types of damage introduced in the specimen structure. The following pairs of sensors were used in the study:

- S1–S4, 285 mm apart from each other;
- S1–S3 and S2–S4, 215 mm apart from each other.

**Figure 3:**
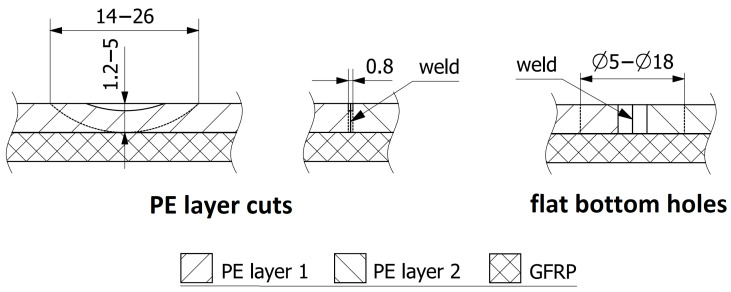
Damage introduced in the PE layer.

**Figure 4:**
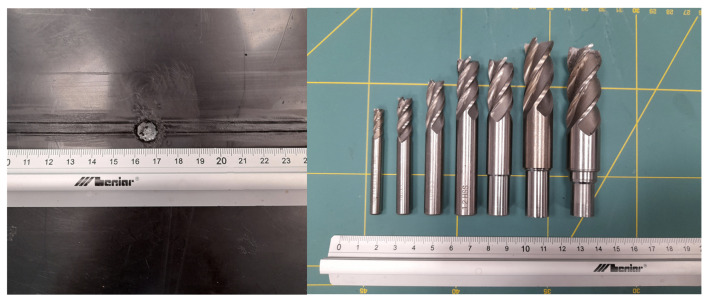
Example of flat bottom hole damage of PE layer and drills used for damage introduction.

**Figure 5:**
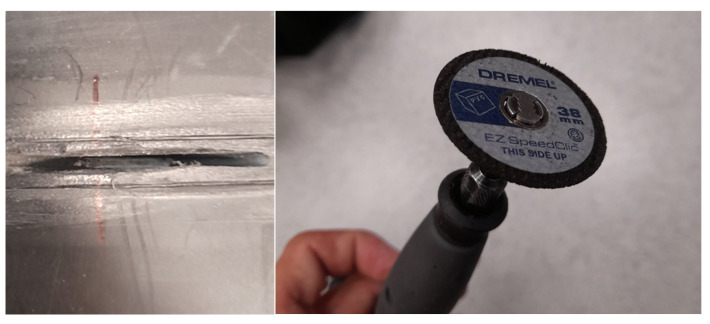
Example of cut made in PE layer and cutting blade used for damage introduction.

**Figure 6:**
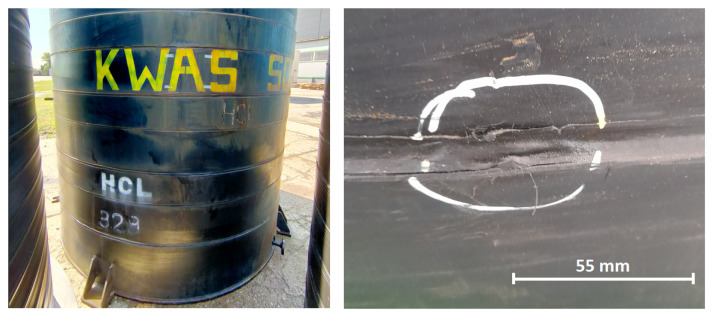
Chemical storage tank for concentrated hydrochloric acid and a crack found in weld of PE layers.

The specimen was subjected to the following types of damage, represented in Figure 3:

1. Flat bottom holes were drilled into the thermal weld of the PE layer, with depths of 5 mm (100% of the PE layer thickness), subsequently increasing in diameter (Figure 4): 5 mm, 8 mm, 10 mm, 12 mm, 14 mm, 16 mm, 18 mm. This type of model damage was intended to simulate the erosion of the PE layer caused by aggressive chemical agents. In a similar way, corrosion loss in metals is often represented in the field of non-destructive testing and structural health monitoring [35,36,37].
2. Cuts were made in the thermal weld of the PE layer with different depths (Figure 5): 25%, 50%, 75%, and 100% of the PE layer thickness. This type of damage aimed to represent cracks in the thermal weld of the PE layer, which could occur due to thermal and load cycles or relaxation of residual stresses. The lengths of the cuts ranged from 14 to 26 mm (Figure 3) and were associated with the cut depth and the diameter of the cutting blade used (Figure 5). Cracks of comparable sizes were observed in chemical storage tanks made of PE material during their operation (Figure 6).

A verification of PZT sensors in the active mode, i.e., with PZT sensors used as a source of elastic waves, was performed on a flat panel similar to the wall section of a large chemical storage tank. Since thick GFRP laminates feature significant attenuation of Lamb waves [38], the adoption of such simplification was justified, and it allowed for an easy and controlled introduction of artificial damage in the PE layer.

In order to verify the efficiency of PZT sensors in passive acoustic emission mode for continuous detection of impacts and AE events related to damage development, composite model tanks were used for pressure tests. These tanks consisted of two layers made of PE and GFRP materials, similar to the flat specimen used for active testing. The GFRPs used for the production of the tanks were manufactured using unidirectional fabric with universal sizing and an areal weight of 500 g/m^2^ (Grm-systems, Olomouc, Czech Republic). Derakane Momentum 470-300 (Ashland’s, Wilmington, DE, USA), a novolac-based epoxy vinyl ester resin, was used as the polymer matrix. It features low viscosity of 325 mPa·s, and a density of 1.08 g/cm^3^. Butanox M-50 methyl ethyl ketone peroxide (MEKP) (Akzo Nobel Functional Chemicals B.V., Amersfoort, The Netherlands) and cobalt naphthenate (CoNap6%) (Sigma-Aldrich, Burlington, MA, USA) were used to initiate the polymerization process in resin. Polyethylene sheets (Röchling SE and Co. KG, Mannheim, Germany) were used as the second layer and thermoplastic liner of the tanks. SE1200 single-end Type 30™ roving from Owens Corning Composite Materials, Toledo, OH, USA and DERAKANE MOMENTUM 470-300 resin were used to manufacture the tank by filament winding.

**Figure 7:**
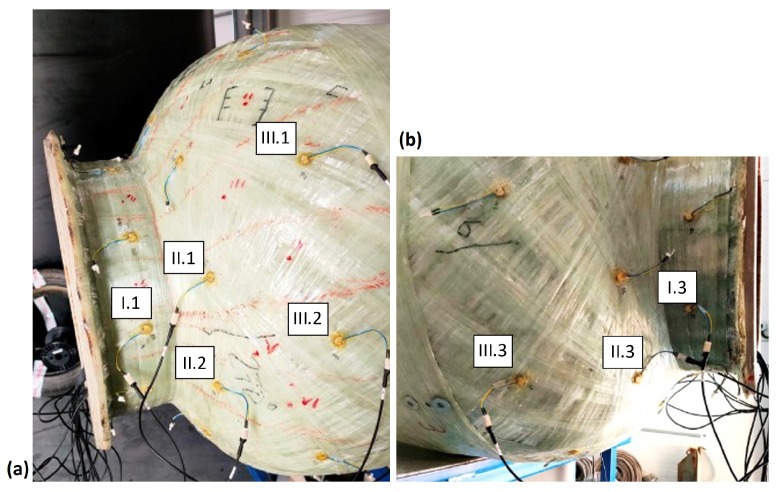
Configuration of the sensor network used for impact testing: (**a**,**b**) shows the opposite sides of the tank.

**Figure 8:**
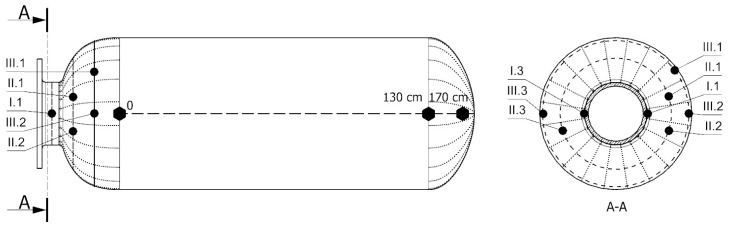
Test tank with indication of sensors localization and the line along which impacts were introduced. Dotted lines on the end caps represent welded joints in the PE layer.

The experiment involved the use of two model chemical storage tanks, each 2 m in height and 0.7 m in diameter. Piezoelectric sensor networks were installed on both tanks. In both cases, the monitored area of the tank was the connection between the cylindrical part of the tank and the flange area (Figure 7). This area is particularly important considering the numerous welded joints in the inner PE wall (Figure 8), potential stress concentrations in the GFRP outer layer caused by variations in the diameter during subsequent glass fiber winding (Figure 7b), and an increased risk of damage from loads applied by screw connections to the flange.

The first tank, used for impact testing and passive acquisition of acoustic signals by PZT sensors, was equipped with a network of Steminc SMD10T2R111WL piezoelectric transducers with the diameter of 10 mm and the thickness of 2 mm. The sensors were distributed circumferentially in one cross section on the nozzle (Section I) and in two additional cross sections on the end cap (Section II and III). For impact testing purposes, eight transducers were selected, which were placed on both sides of the flange, as shown in the illustrations below (Figure 7 and Figure 8).

**Figure 9:**
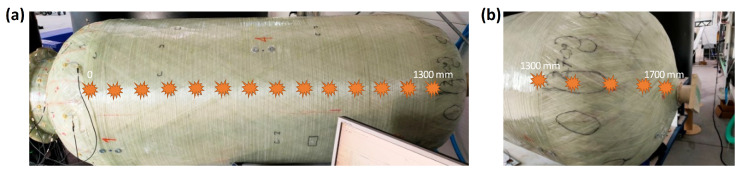
Locations where impacts were introduced: on (**a**) the shell and (**b**) the end cap.

In the research, the amplitude distribution of signals was analyzed as a function of the force and the impact distance along the tank axis. Impacts were introduced at 100-mm intervals, starting from the joint between the end cap and the shell (Figure 8 and Figure 9). At each point, at least 100 impacts were introduced with varying levels of impact force ranging from 3 to 150 N. The angular orientation of the line along which impacts were introduced corresponded to the orientation of the I.1 sensor and was opposite to the orientation of the I.3 sensor (Figure 7 and Figure 8).

**Figure 10:**
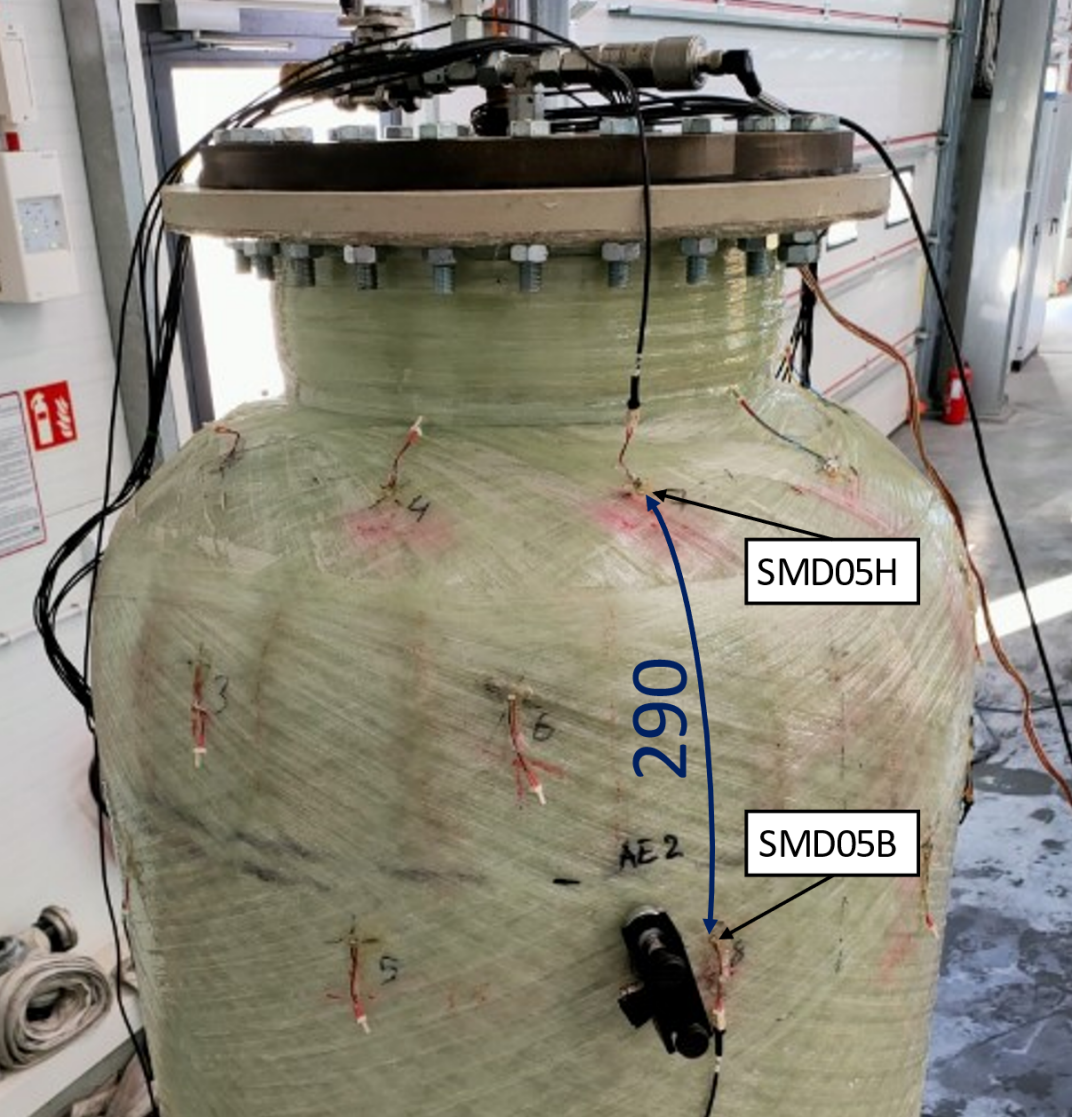
Configuration of the sensor network used for pressure testing.

The second tank was used to compare the efficiency of different types of PZT sensors during a pressure test. Three sensing networks were installed on the tank’s surface, differing in types of STEMINC Inc. sensors used: SMD05T04R111WL, SMD07T05R411WL, and SMD10T2R111WL, each with different dimensional and electrical parameters. The network layout for each type of sensors was the same, and different networks were distributed circumferentially. During the pressure test, continuous signals were measured from six sensors—two from each network, located in two cross sections in the flange and shell areas, as shown in Figure 10 below for the SMD05T04R111WL sensor type.

The pressure test was conducted by gradually filling the tank with water in five steps differing in pressure: 0, 2, 4, 6, and 9 bar. Water was used as a test medium for safety reasons, considering the possibility of a leakage during the test. However, no significant loss in generality of the obtained results was expected, as water provides equivalent load conditions for acoustic emissions in the pressure test. Some difference in signals with respect to leakage or pumping can occur due to variations in the density and viscosity of water compared to typical media stored in chemical storage tanks. Between each pressure change, a minimum period of ten minutes was maintained at a stabilized pressure level. During the last step at 9 bar, a crack in the internal PE layer of the tank wall emerged, which resulted in fluid leakage onto the tank’s surface and the termination of the test.

### 2.2. Data Acquisition from PZT Sensors

**Figure 11:**
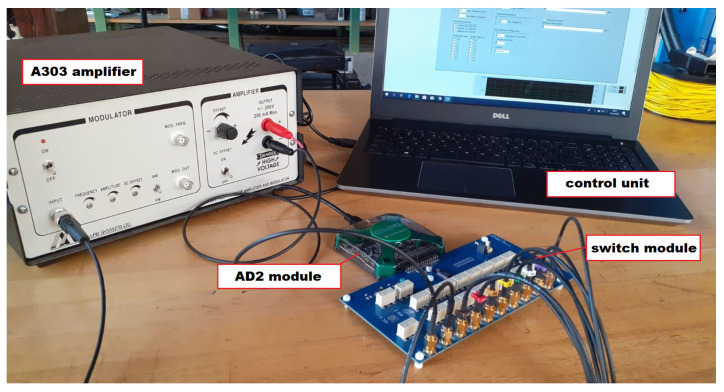
The acquisition system used in the experiment.

For sensors excitation and signal acquisition, a dedicated system was used, based on the Analog Discovery 2 (AD2) module by Digilent [39] connected with an eight-channel relay-switch module (Figure 11). The signal generator was connected to the A303 high voltage amplifier [40] to generate a 100 Vpp (symmetric) excitation signal. For pulsed excitation, the following parameters of signal were used:

- Excitation frequencies [kHz]: 100–360 kHz with a 20 kHz increment;
- Excitation window: Hanning;
- Number of periods of the excitation signal: 3, 8.

Additionally, harmonic excitation of PZT sensors in the frequency range of 100–350 kHz with 1 kHz increment was applied.

**Figure 12:**
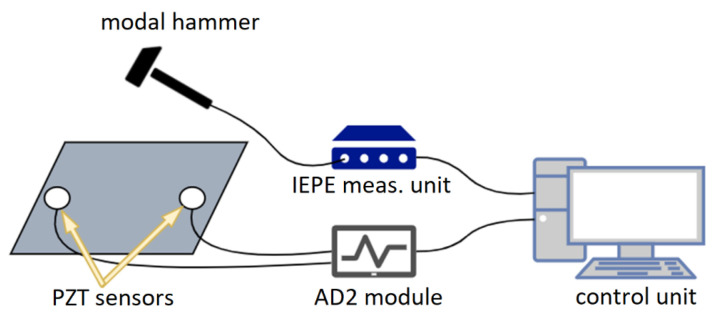
The developed measurement setup for impact testing.

The measurement system was also adapted to acquire acoustic emission signals. It was used to evaluate the effectiveness of PZT sensors in detection of impact events and acoustic emission signals excited during the pressure testing of the vessel. For the impact testing experiments, the system was supplemented with a modal hammer equipped with an IEPE-type accelerometer sensor, calibrated to measure the force of the applied impacts (Figure 12). The readings from this sensor were acquired by the NI 9230 measurement module with a minimal force sensitivity of 3 N.

In the pressure testing, three Digilent Analog Discovery 2 systems were used, connected in parallel. This configuration increased the number of independent measurement channels to six. Acoustic emission signals were continuously recorded at a sampling frequency of 250 kS/s.

## 3. Results and Discussion of the Experiments

### 3.1. Application of PZT Sensors for Damage Detection of PE Layer

Guided waves are elastic waves that propagate through a structure, “guided” by its physical boundaries, known as Lamb waves for thin plate-like structures [41]. They can propagate over long distances for the sensor’s size, which makes them well suited for the monitoring of large structures. Structure monitoring with use of Lamb waves involves introducing these waves into the structure and then capturing them, often at multiple locations. Both tasks can be accomplished using a network of PZT transducers integrated with the monitored structure [25]. Lamb waves are multimodal, i.e., for a given frequency, at least two fundamental modes of Lamb waves with different propagation speeds can be simultaneously excited in the structure. Furthermore, since they propagate over significant distances, they can scatter on natural structure discontinuities and reinforcements, which makes the analysis of signals acquired by PZT sensor networks particularly challenging.

Therefore, due to their high complexity, signal changes are often evaluated based on the so-called damage indices (DIs). These indices are numerical characteristics that contain information about energy change in a given signal or its redistribution. This makes them sensitive to potential signal changes caused by damage, such as amplitude drop, or local amplitude changes due to transmission and reflection of elastic waves from structural discontinuities. In this study, the following signal characteristics, which are often used for structural health monitoring, were investigated [25,26]:

(1) $$\begin{array}{cc} \; & corr=1-r_{f_{gs},f_{gs,b}}\\ \; & envCorr=1-r_{f_{gs}^{env},f_{gs,b}^{env}}\\ \; & sigDiffRMS=\sqrt{\frac{\int_{T}{\left(f_{gs}-f_{gs,b}\right)}^{2}dt}{\int_{T}f_{gs,b}^{2}dt}}\\ \; & diffRMS=\sqrt{\left|\frac{\int_{T}f_{gs,b}^{2}dt-\int_{T}f_{gs}^{2}dt}{\int_{T}f_{gs,b}^{2}dt}\right|}\\ \; & divAmp=\left|\log\left(\frac{\max_{t\in T}}{\max_{T}}\right)\right| \end{array}$$

where $f_{gs}$ denotes the signal captured by a PZT sensor s and which is induced by propagation of elastic wave actuated by PZT transducer *g*, $f_{gs}^{env}$ is the envelope of the signal $f_{gs}$, $f_{gs,b}$ and $f_{gs,b}^{env}$ denote the corresponding baseline signal and its envelope, and $r_{x,y}$ denotes the correlation coefficient between signals *x* and *y*. The above signal characteristics are sensitive to both amplitude and phase changes in baseline signals. In addition, these characteristics differ in the information content carried by the captured signals. The damage indices corr, envCorr, sigDiffRMS are sensitive to local changes in the signal, both in the amplitude and in the signal phase (corr and sigDiffRMS only). The damage indices diffRMS and divAmp provide limited information about the signal, i.e., about the total energy and maximum amplitude, respectively.

Modeling the propagation of Lamb waves and their interaction with damage is alone a large research area. Even in the simplest case of Lamb wave propagation in an isotropic homogeneous medium, exact closed-form solutions to the equations of linear elastodynamics leads to implicit form of dispersion relations [25,41], which can only be solved numerically. Therefore, since the early stages of PZT sensor application for structural health monitoring, the development of numerical and analytical tools for a better understanding of elastic wave propagation in thin-walled structures and their interaction with damage, such as cracks or cylindrical cavities, has been of utmost importance [42]. Over the years, numerous approaches to this topic have been proposed [42,43]. The choice of a particular method depends on the trade-off between computational efficiency and accuracy of a given approach, especially when developing numerical models for more realistic structures. Apart from the selection of appropriate tools for the analysis of particular problem, another challenge is characterizing the materials used appropriately, defining all of the contact conditions, e.g., for structure discontinuities caused by damage or naturally present adhesive bonds of sensors with the substrate material, or when some structure reinforcements are present. Furthermore, additional factors can contribute to signals captured by PZT sensors, e.g., properties of the particular PZT ceramic, the true distribution of the electric field used for sensors excitation, external measurement conditions, or the characteristics of electronic devices used for signal acquisition. These factors make the problem of signal reconstruction and the assessment of DIs change due to the occurrence of damage even more challenging.

This paper focuses on an anisotropic GFRP material with a bonded viscoelastic PE layer. Damage to a finite extent, i.e., cylindrical cavities and cuts simulating open cracks, was introduced in the thermal weld of PE layer. Notably, none of the damage ran through the entire thickness of the specimen, which would cause additional complexity for modeling. Since the described case of structural health monitoring is not common, there are no studies specifically devoted to the types of the structure considered in this paper. However, some effects can be predicted based on research findings that partially correspond to the considered damage scenarios.

Significant efforts have been made to develop theories and numerical tools for modeling the propagation of guided waves in layered anisotropic media, in particular in composite materials such as the GFRP layer in a chemical storage tank’s wall structure [28,44,45,46,47], also bonded with viscoelastic layers [48]. Combined with the models of elastic wave scattering on joints of plates [49,50], e.g., the thermal weld in the PE layer, the use of these methods could provide a reliable basis for the simulation of guided wave propagation in undamaged structures. One of the fundamental mechanisms of elastic wave interaction with damage is forward and backward scattering, both in the case of introduced cuts and simulated erosion of the PE layer [51,52,53,54,55,56,57]. The amplitude of transmitted and backscattered waves depends on various factors, including excitation frequency (which determines the relative length of the excited Lamb waves and the extent of damage) and damage depth [53]. It is worth noting that transmission and reflection coefficients, and consequently also amplitude-dependent damage indices, may not always depend monotonically on the size of damage [53,57]. Among the considered damage indices (as defined in Equation (1)), the divAmp, sigDiffRMS, and diffRMS signal characteristics should be particularly sensitive to changes in the amplitude of incident waves as they are transmitted through the damage. In general, also waves reflected from specimen boundaries or scattered on reinforcements can contribute to the signal received by the PZT sensor; therefore, in such a case, signal characteristics based on correlation coefficient corr, envCorr can also be sensitive to changes in signal amplitude. These characteristics can also be applied when the transmission of incident waves through the damage alters their phase [56]. Another effect in the acquired signals following damage can be the emergence of new components due to elastic wave modes conversion, radiation of Rayleigh-like waves propagating along the edges of the damage, or the excitation of creeping waves [52,56,58,59]. Such effects can be captured in particular by phase-sensitive damage indices like corr, sigDiffRMS, or by the envCorr signal characteristic.

**Figure 13:**
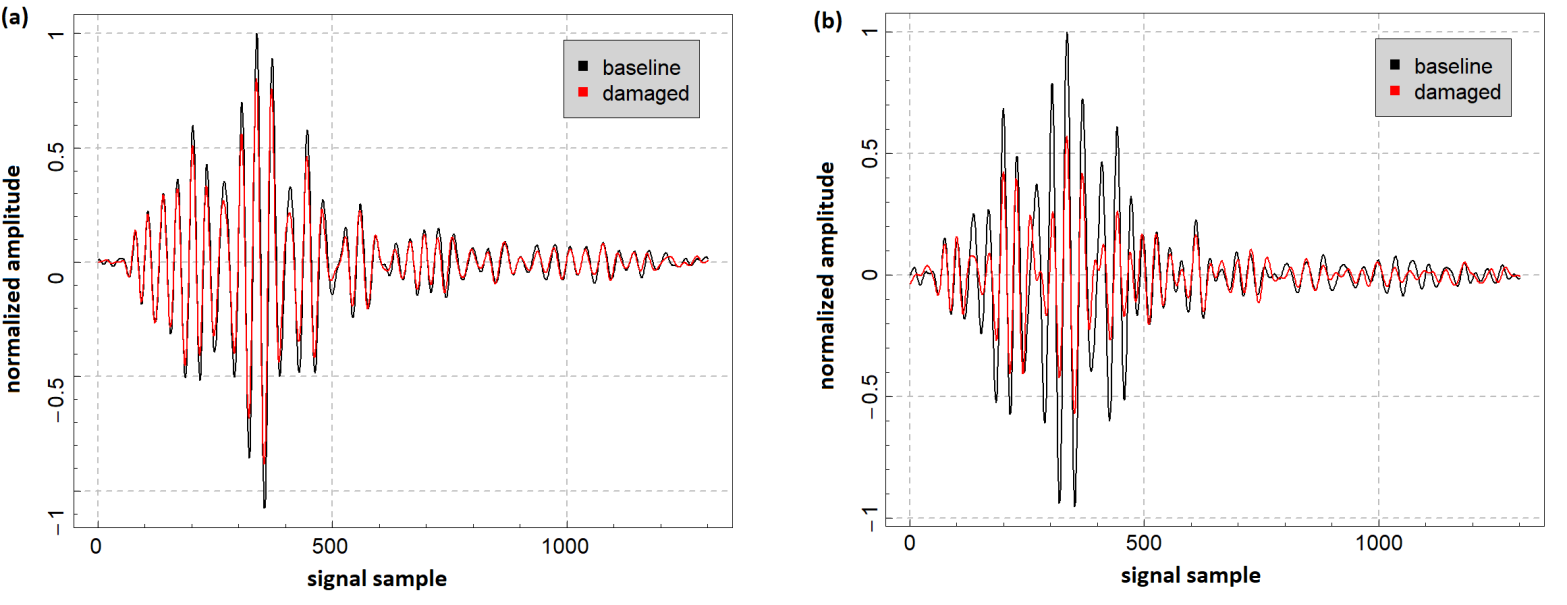
Example of normalized signals captured by a PZT sensor for the pristine state of the structure and: (**a**) 100% in thickness cut introduced in the PE layer and (**b**) 18 mm in diameter flat bottom hole drilled in the PE layer.

In Figure 13, examples of normalized signals acquired by a PZT sensor for the pristine state of the structure and after damage introduction in the PE layer are presented. The excitation frequency was set to 260 kHz and the duration of the excitation signal was three periods for all the presented cases. After cutting the PE layer (Figure 13a), there was a noticeable drop in the amplitude of the dominant signal component; however, neither signal phase changes nor signal energy redistribution were caused by this damage. Amplitude-sensitive DIs such as divAmp, diffRMS, or sigDiffRMS should be suitable in such cases. However, since amplitude changes do not occur uniformly across the entire time domain of the signal, corr and envCorr signal characteristics can also capture this effect to some extent. When Lamb waves interact with a flat bottom hole introduced in the PE layer, less uniform amplitude variation with respect to the baseline signal can be observed (Figure 13b). Additionally, there are also noticeable signal phase disturbances at specific instances of time. In this scenario, the DIs corr, sigDiffRMS, and envCorr are well suited for the detection and tracking of such effects.

Since different damage indices do not scale in the same way with respect to damage or other measurement conditions, their bare values cannot be directly compared. Therefore, in this experiment, two sets of the defined damage indices (corr, envCorr, sigDiffRMS, diffRMS, diffAmp, divAmp) were calculated for every combination of excitation parameters. One set was based on two reference signals acquired when the structure was in its pristine state. These damage indices are denoted as $DI_{ref}$. This set allows to determine the level of change in damage indices due to variations in acquired signals related to measurement noise under laboratory conditions. Additionally, based on signals acquired after damage introduction and one of the reference measurements set, damage indices $DI_{dam}$ corresponding to damage development were calculated. For data analysis, it is convenient to use relative damage index values, defined as the ratio:

(2) $$DI_{rel}=\frac{DI_{dam}}{DI_{ref}},$$

as those provide more direct information about the possibility of damage detection in a given case, which allows for an efficiency comparison of different damage indices. As a threshold for the system’s applicability, based on data provided by a manufacturer of chemical storage tanks, it was assumed that the changes in damage indices due to damage should be at least 10 times greater than the changes caused by measurement noise under repeatability conditions in the laboratory.

**Figure 14:**
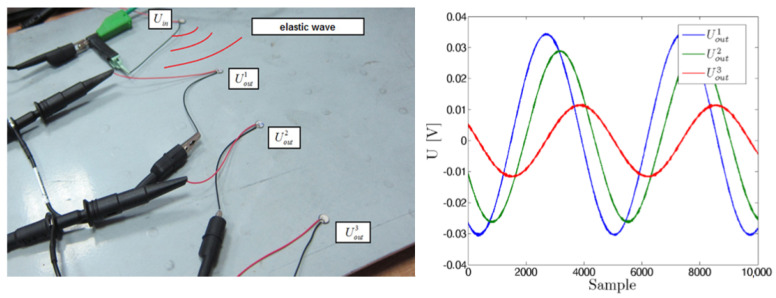
Example of signals captured by three different PZT sensors for harmonic excitation [60].

In addition to pulsed excitation of PZT sensors, which provides signals confined to a specific time period, harmonic excitation of PZT sensors was also applied. In this case, the signals acquired by PZT sensors are harmonic with the same frequency as the excitation signal, but differ in the phase and the amplitude as shown in Figure 14 below.

In this case, the voltage transfer ratio method for signal analysis was applied. As described in [60,61], this method relies on the following damage indices definition:

(3) $$DI\left(\omega \right)=\frac{U(\omega )}{U_{0}\left(\omega \right)}\exp\left(i\delta \phi \left(\omega \right)\right),$$

where U(ω), $U_{0}\left(\omega \right)$ denote the amplitude of the harmonic signal captured by a given sensor and the corresponding baseline signal for the frequency of the excitation ω and δϕ(ω) denotes the phase difference between the captured and the corresponding baseline signal (which also depends on the excitation frequency). In an undamaged state, these DIs are concentrated in the vicinity of the point 1+i0 in the complex plane, irrespectively of the excitation frequency. If damage is present, it can alter both the output voltage amplitude and its phase, similarly as in the case of pulsed excitation. Therefore, such DIs can deviate from the point 1+i0, particularly for frequencies that are sensitive to damage [60,61].

For harmonic excitation, the obtained DIs were filtered to retain only the central 50% of the data points, closest to the median point within a given dataset. The ratio between traces of the covariance matrix obtained for the DIs set corresponding to damaged state of the structure and the covariance matrix of data obtained under repeatability conditions in the laboratory was adopted as relative damage index in that case. Therefore, the relative damage index for the harmonic excitation is defined as follows:

(4) $$DI_{rel}=\frac{Tr(Cov\left(DI_{dam}\right))}{Tr(Cov\left(DI_{ref}\right))}.$$

This signal characteristic measures the difference in variance of data obtained for the damaged and undamaged state of the structure.

#### 3.1.1. Detection Efficiency of Flat Bottom Holes Introduced in PE Layer

**Figure 15:**
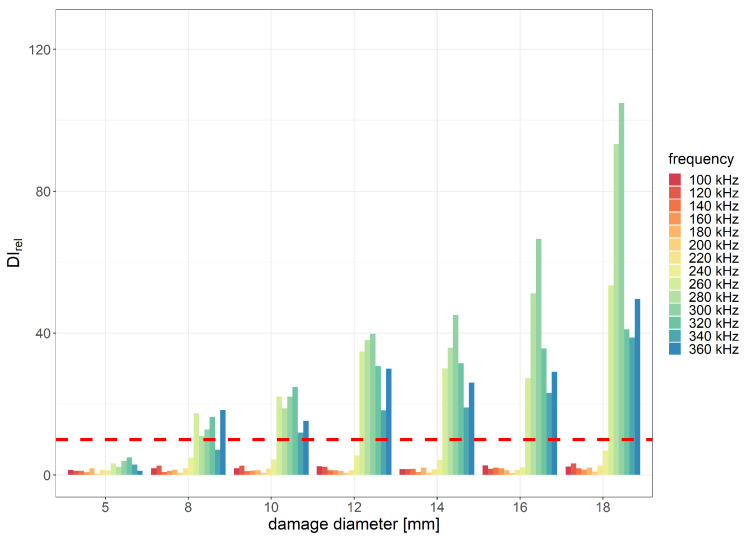
Relative envCorr damage index values obtained for flat bottom holes of PE layer with indication of the assumed efficiency threshold level for the distance between PZT sensors 215 mm and 8 periods of the excitation signal.

**Figure 16:**
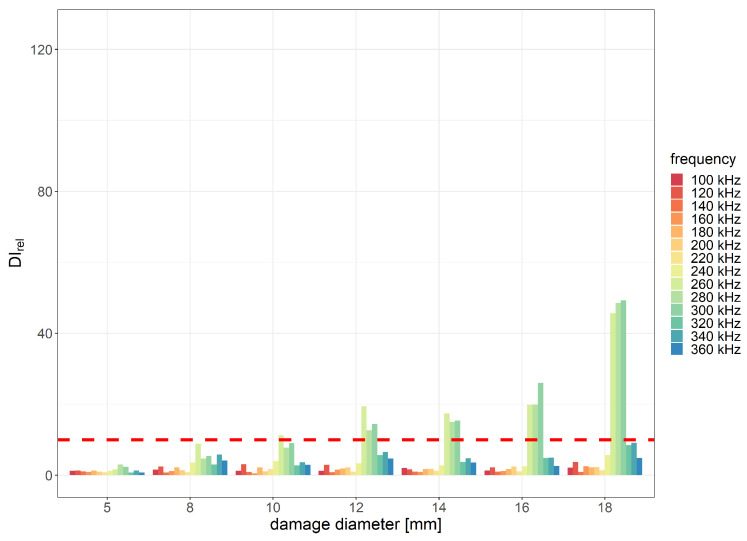
Relative envCorr damage index values obtained for flat bottom holes of PE layer with indication of the assumed efficiency threshold level for the distance between PZT sensors 285 mm and 8 periods of the excitation signal.

For flat bottom holes introduced in the PE layer, the best relative damage index values were obtained for the envCorr and corr signal characteristics for eight periods of the excitation signal. Relative values of envCorr damage index with indication of the assumed threshold level for the two distances between the sensor and the generator are shown in Figure 15 and Figure 16 below.

The most efficient range of frequencies for damage detection usually depends on several factors, including the type and size of the damage, properties of the sensors used, as well as material properties of the monitored structure. In the case considered in this study, where the material consists of two layers with significantly different elastic properties, the excitation frequency can affect the amplitude of guided waves that can pass the interface between GFRP and PE layers. In the experiment, the best damage detection capability was achieved within the frequency range of 240 to 320 kHz. The minimum diameter of flat bottom holes that met the predefined applicability requirements in terms of the $DI_{rel}$ threshold was 8 mm for the distance between PZT sensors equal to 215 mm and 12 mm for the distance equal to 285 mm. For the damage diameter of 18 mm, the obtained envCorr value caused by damage compared to noise influence was approximately 100 times higher for the distance 215 mm between PZT transducers and about 50 times higher for the of 285 mm between sensors.

**Figure 17:**
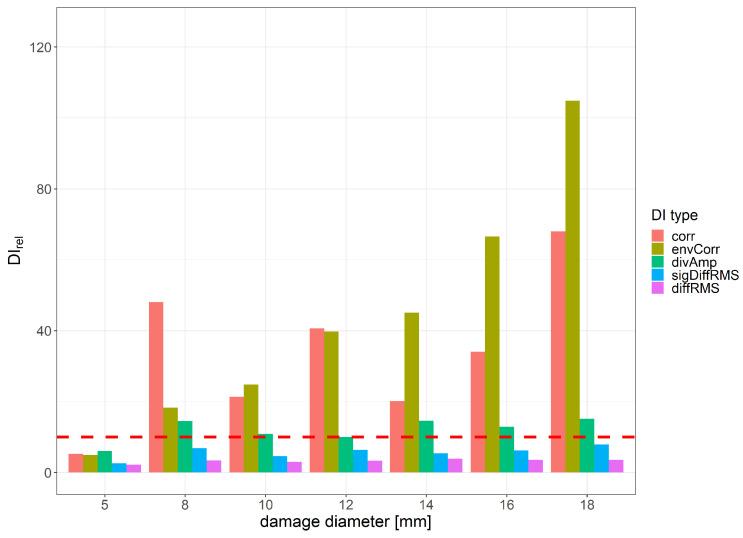
Maximum value of relative DI change for 8 periods of excitation signal with respect to damage diameter.

For the envCorr signal characteristic, a monotonic relationship with damage size was observed, whereas the corr DI was saturated for the damage size of 8 mm, as shown in Figure 17, which presents the maximum relative values of different DIs with respect to damage size. The efficiency of damage detection for small damage was the highest for the corr DI, while for damage with diameters greater than 14 mm, the efficiency of envCorr DI surpassed other DIs. The assumed threshold level for damage detection efficiency was also obtained for corr DI. Among DIs carrying low information content about the signal, the damage index was based on amplitude comparison, i.e., the divAmp signal characteristic was more efficient than diffRMS, which relies on signal energy comparison. Additionally, the divAmp characteristic outperformed sigDiffRMS, which is designed to be sensitive to a wider range of signal changes.

**Figure 18:**
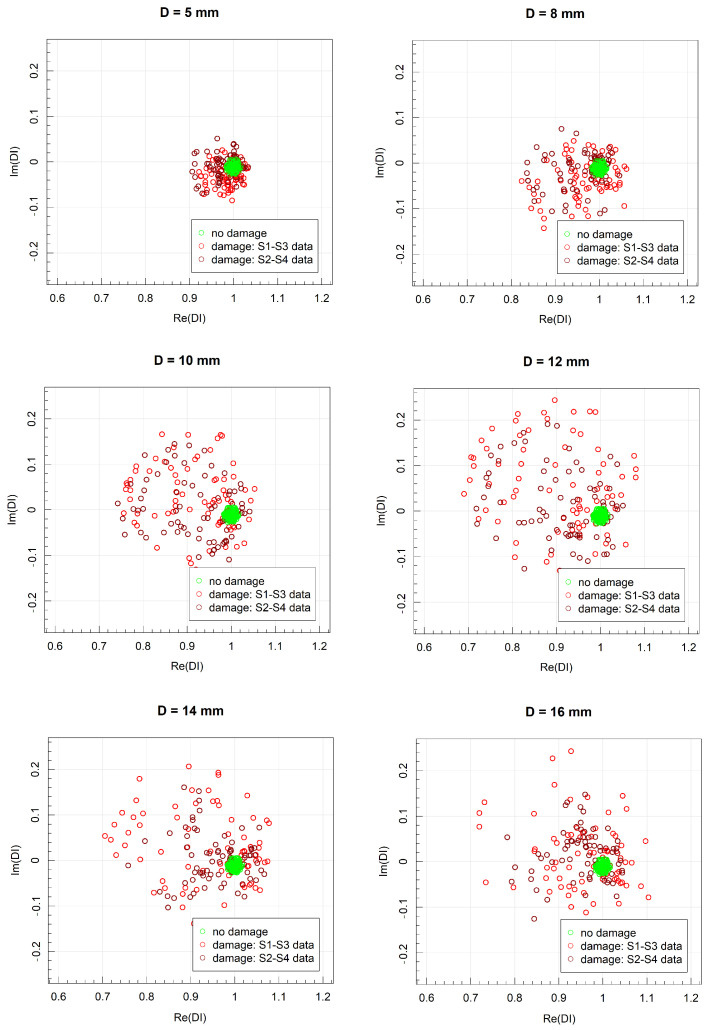
DIs values obtained for harmonic excitation of PZT sensors for different diameters of damage for distance between sensors equal to 215 mm.

**Figure 19:**
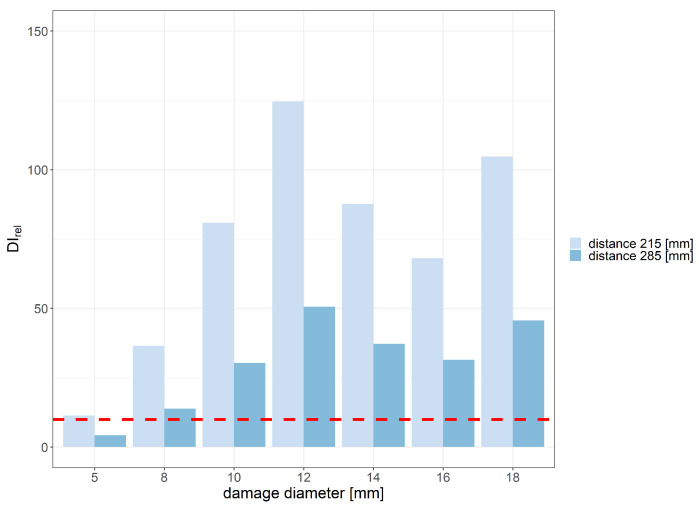
Relative damage index values for harmonic excitation obtained for different damage diameters and distances between sensors.

In Figure 18, the DIs’ values obtained for harmonic excitation of PZT sensors with respect to different diameters of damage for distance between sensors equal to 215 mm are represented in the complex plane. With the initial increase in damage diameter, both a greater spread of data as well as the shift from the point 1+i0 can be observed. This effect stabilizes after reaching the damage diameter of 12 mm. The relative change in DIs, measured as the ratio between data spread for the undamaged and damaged states of the structure, is presented in Figure 19. The assumed threshold level for damage detection efficiency for this type of damage indicator was obtained for the damage diameter exceeding 8 mm for both distances between the generator and the sensor.

#### 3.1.2. Detection Efficiency of Cuts Introduced in PE Layer

**Figure 20:**
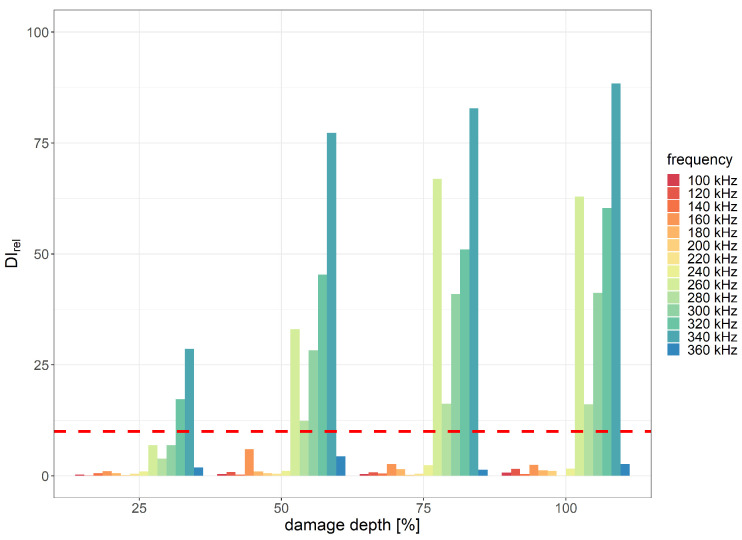
Relative divAmp damage index values obtained for cuts of PE layer with indication of the assumed efficiency threshold level for the distance between PZT sensors 215 mm and 3 periods of the excitation signal.

**Figure 21:**
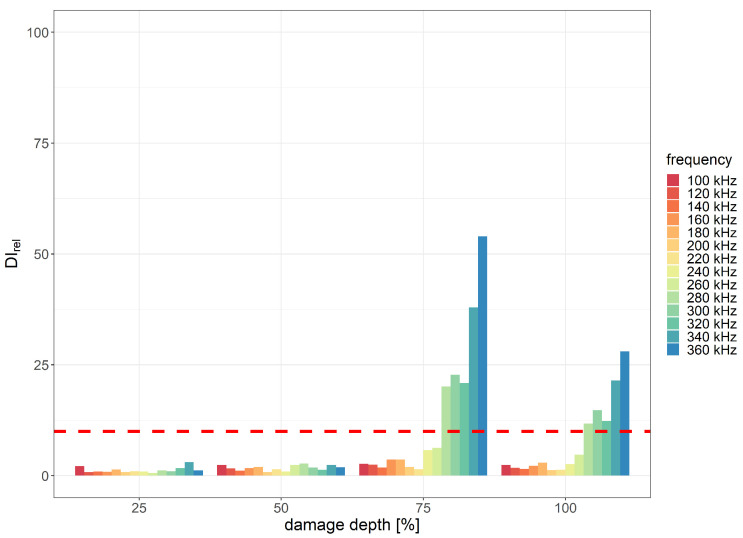
Relative corr damage index values obtained for cuts of PE layer with indication of the assumed efficiency threshold level for the distance between PZT sensors 285 mm and 8 periods of the excitation signal.

In the case of cuts of thermal weld introduced in the PE layer, the highest damage detection efficiency was obtained within the excitation signal frequency range of 240–360 kHz for two scenarios:

1. The divAmp damage index exhibited the best performance with the excitation signal duration of three periods for the distance between sensors equal to 215 mm (Figure 20);
2. The corr damage index was the most efficient for eight periods of the excitation signal for the distance between sensors equal to 285 mm (Figure 21).

**Figure 22:**
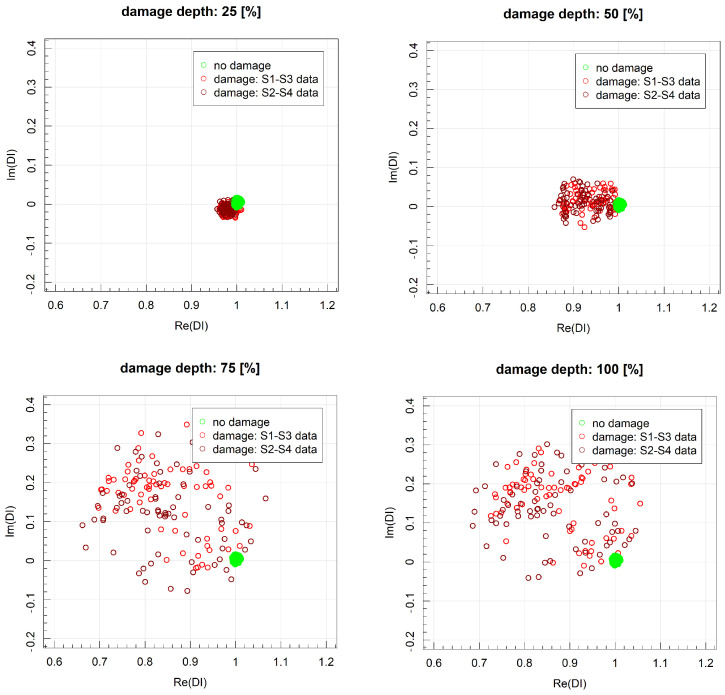
DIs values obtained for harmonic excitation of PZT sensors for different depths of damage for distance between sensors equal to 215 mm.

**Figure 23:**
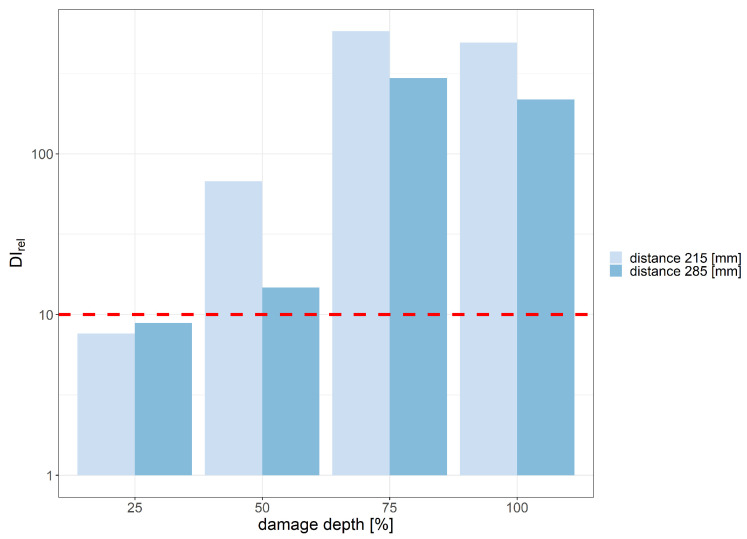
Relative damage index values for harmonic excitation obtained for different damage depths and distances between sensors.

The minimum depth of the PE layer cut, for which applicability requirements with respect to $DI_{rel}$ threshold were met, was 25% of the layer thickness for the distance between PZT sensors equal to 215 mm and 75% of the layer thickness for the distance equal to 285 mm. Th damage detection efficiency of other DIs was significantly lower compared to divAmp and corr signal characteristics. In Figure 22, the DI values obtained for harmonic excitation of PZT sensors with respect to different cut depths for distance between sensors equal to 215 mm are represented in the complex plane. Similarly, as in the case of flat bottom holes, an increase in cut depth led to both greater spread of data as well as deviation from the point 1+i0. The relative change in DIs, measured as the ratio between data spread for the undamaged and damaged state of the structure, is presented in Figure 23. The assumed threshold level was achieved for damage depth exceeding 50% of the PE layer thickness for both distances between sensors. When the depth of damage exceeded 75% of the layer thickness, the spread of damage index data corresponding to the damaged state of the structure exceeded the spread of data due to signal noise under the laboratory conditions by 100 times.

### 3.2. Application of PZT Sensors for Passive Detection of Impacts and Acoustic Emission Events

**Figure 24:**
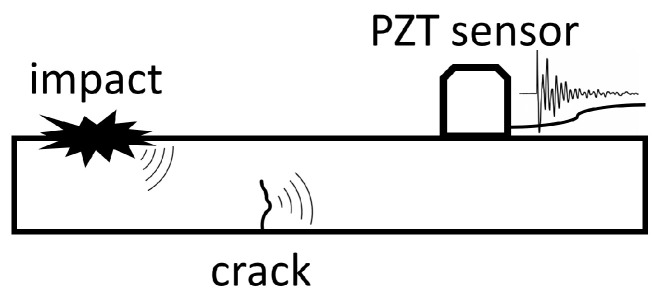
Passive detection of signals generated by impact and cracks by PZT sensors [69].

In this study, PZT sensors were also used in the passive manner for the acoustic emission (AE) method [62,63,64,65,66,67] or impact events detection. In this approach, the sensors serve as receivers of elastic waves generated in the material due to rapid local stress redistribution. These waves can arise from impacts or the initiation and progression of damage in composite structures, including phenomena such as matrix cracking, fiber breaking, and debonding [64,68], as illustrated in Figure 24.

In the passive mode of operation, PZT sensors can effectively detect propagating waves when events like impact or composite fiber breakage occur, similarly as waves actively excited by PZT actuators. However, unlike active methods, the passive measurements mode cannot be repeated for a specific structural state. To generate new acoustic emission signals, further damage development is required. This requires continuous data acquisition and limits the ability for inferring changes in the structural state.

However, the passive mode of PZT sensors operation can be successfully used as a source of valuable information regarding events such as impacts, which can be further investigated in greater detail using active methods or non-destructive testing. Additionally, AE signals can be also used to determine localization of the source of elastic waves, such as developing damage or impact event. With an appropriate signal database and the development of suitable algorithms, AE signals could enable the classification of events into different classes of damage of the monitored structure or impacts events [66].

**Figure 25:**
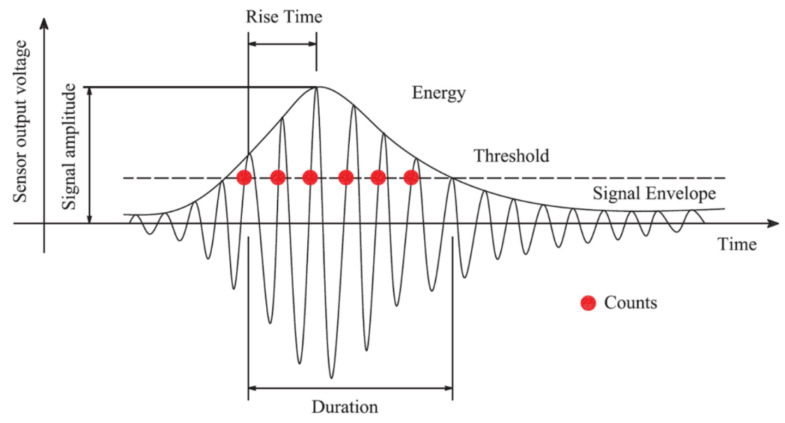
Defined parameters of acoustic-emission events [67].

For the analysis of acoustic emission events, a series of signal characteristics has been defined [62]. A graphic representation of these parameters is presented in Figure 25 in relation to an example of a model acoustic emission signal.

In impact testing, the amplitudes of sensor responses to the applied impacts were examined with respect to impact’s location and force. The obtained data were compared for sensors positioned at different cross sections and peripheral locations to determine the optimal sensor distribution within the target tank monitoring system. The analysis was conducted based on the signal-to-noise ratio (SNR), which, according to the considered applicability requirements, should be at least 10.

During the pressure test, the continuously recorded signals from the sensors were processed to detect the envelope of acoustic emission impulses, enabling further analysis of specific events. Due to significant differences in noise levels among the measurement channels, individual triggering thresholds were adopted for each of them based on two SNR levels: 1.5 for initial event searching and 10 as the target threshold, in accordance with the determined requirements. To compare the measurement capabilities of the three sensor types, the number of identified acoustic emission events was determined for each sensor. Additionally, characteristic parameters for the same events recorded by different sensors were also calculated.

#### 3.2.1. Detection Efficiency of Impacts

**Figure 26:**
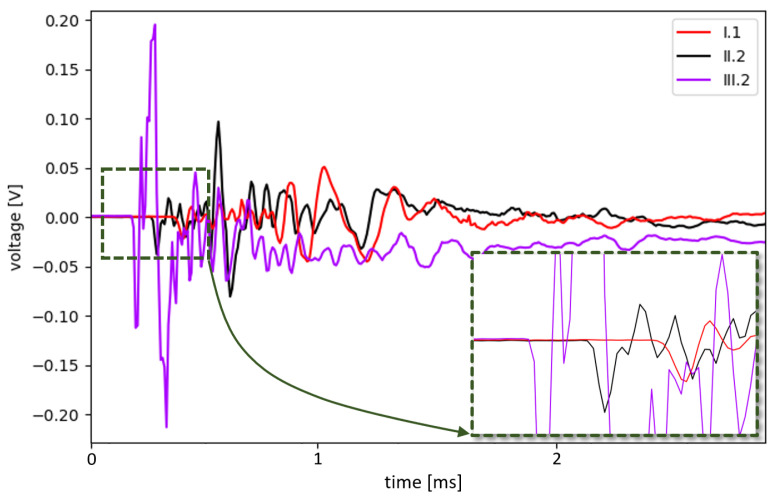
Examples of signals acquired by PZT sensors due to an impact.

The readings from all the sensors demonstrated the ability to detect impact events throughout the entire tank, including the distant end cap, which includes an inner PE layer composed of separately welded elements (Figure 8). In Figure 26, examples of signals acquired by PZT sensors due to an impact event are presented. The distance to the impact point varied for the three considered sensors: the sensor labeled III.2 was located closer, and the sensor labeled I.1 was positioned further from the impact point than the other sensors, respectively. A time delay between signal peaks can be observed for different sensors; also, maximum peak values are decreasing with the distance from the impact point, which are typical signal features corresponding to acoustic wave propagation induced by a point source.

**Figure 27:**
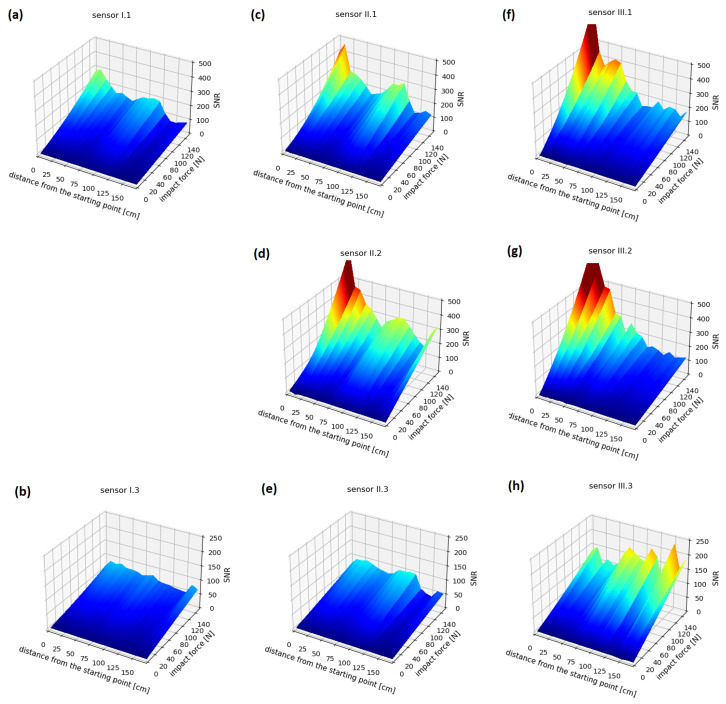
Impact test results: (**a**–**h**) depicts data obtained for subsequent sensors of the network.

In Figure 27, the mean SNR values of signals acquired by sensors with respect to the distance between an impact location and the selected origin (Figure 8), as well as the impact force, are presented. For impacts located in the shell section of the tank, non-monotonic SNR changes with respect to the distance from the origin can be observed for sensor II.1 and sensor II.2, which can indicate presence of local areas of increased damping or stiffness variation. For the sensors labeled I.1 and II.1, impacts in the end cap area (at distances exceeding 1300 mm) resulted in events with significantly lower SNR, decreasing abruptly compared to impacts on the shell. This could be due to the structural differences between the cap and the shell—in particular, the presence of many welded joints in the PE layer of the cap structure (Figure 8).

Dependence of the signal level on the sensor installation location can also be observed. However, the differences between the measurement cross sections are consistent with their distances from the impact locations, with the highest values obtained in cross section III, which is the closest to the selected origin (Figure 8). Significant differences also occurred depending on the location along the circumference of a given cross section. Sensors I.3, II.3, and III.3, located opposite to the line of impacts (Figure 8), generated readings several times lower than the rest of the sensor network. These sensors also exhibited different responses to impacts in the end cap area, where the amplitudes increased compared to the signals recorded from the impacts on the cylindrical shell, which is contrary to the distribution observed for other sensors. This effect can be due to shorter propagation paths of elastic waves on the spherical surface in the end cap compared to the circumferential paths around the cylinder in the shell. For the remaining pairs of sensors located in the same cross sections and near each other (II.1–II.2 and III.1–III.2), the distribution of readings is similar, with differences in amplitudes ranging up to several percent.

**Figure 28:**
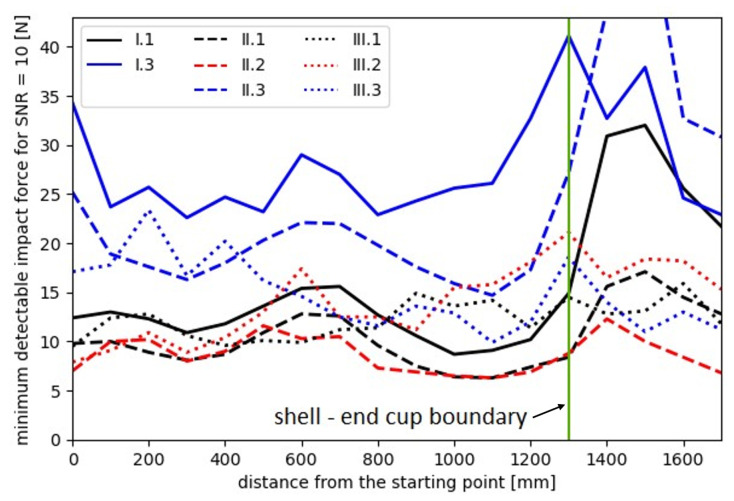
Minimum detectable impact forces at an SNR level of 10.

In addition, Figure 28 presents the lowest levels of impact forces for all sensors that meet the requirement of a signal-to-noise ratio level equal to 10, which helps to determine the minimum impact force that can be reliably detected by each sensor.

#### 3.2.2. Detection Efficiency of AE Events during Pressure Test

**Figure 29:**
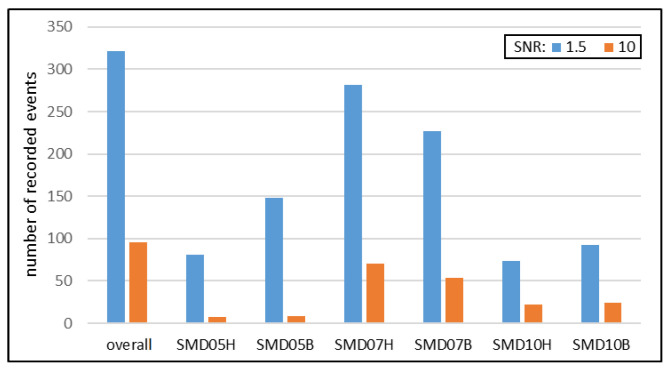
Summary of the number of detected AE events.

During the pressure test, a total number of 321 acoustic emission events were recorded with amplitudes exceeding the noise level by at least 50%. Among these events, 96 events also exceeded the SNR threshold of 10. The numbers of events detected by each sensor are presented in the following diagram (Figure 29).

**Figure 30:**
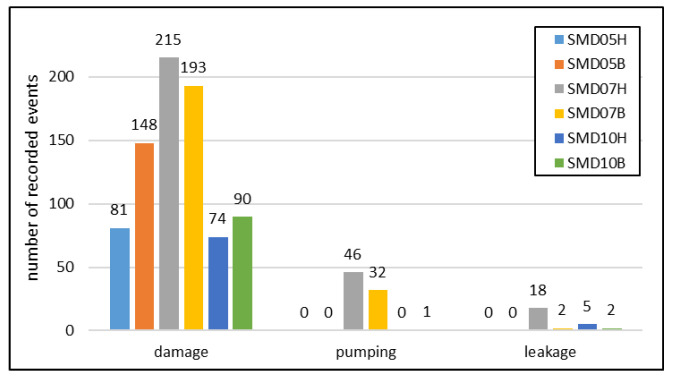
Distribution of the number of AE events depending on the signal source.

**Figure 31:**
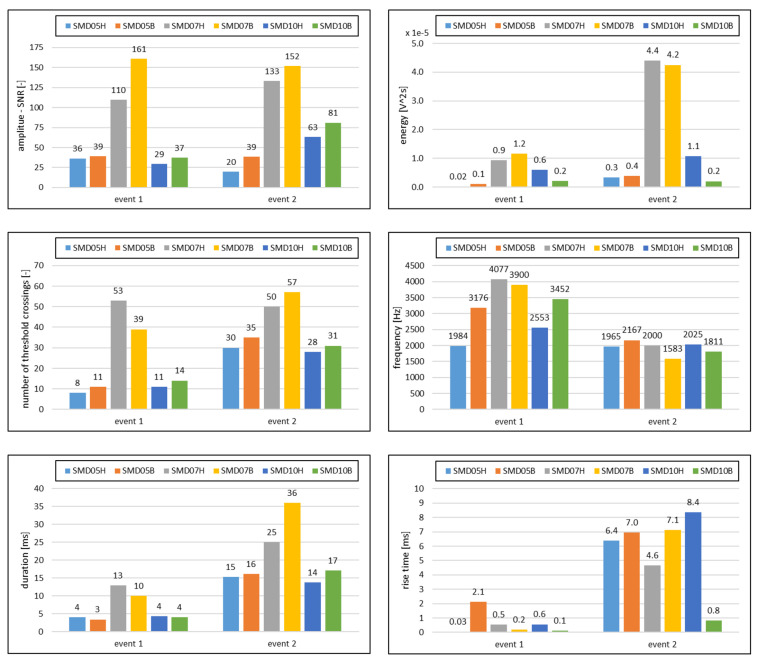
Comparison of sensor response parameters for identical events.

The results confirm the feasibility of using the acoustic emission method with all three types of PZT sensors. However, significant differences in the number (Figure 29 and Figure 30) and the strength (Figure 31) of recorded signals are observed between the sensor types. The Steminc SMD07T05R411WL sensors (SMD07H and SMD07B) recorded the highest number of signals, approximately twice as many as the other two types of sensors (Figure 29). The lowest number of indications with the SNR values exceeding 1.5 was achieved for the Steminc SMD10T2R111WL sensors (SMD10H and SMD10B). However, for these sensors, the number of indications at the SNR equal to 10 is higher than for the third type. This suggests that sensors of that type require higher elastic wave energy as trigger than SMD05T04R111WL, but they also provide stronger responses.

The registered AE events were categorized based on their causes. The summary of the number of signals at SNR exceeding 1.5 is shown in Figure 30. Water pumping processes generated low-frequency and low-amplitude signals, which were distinguishable from high-frequency events related to structural damage. Signals similar to filling of the vessel were also recorded when leaks occurred during the final phase of the test. Such signals are not strictly related to energy release due to the degradation process of the internal structure of the material, however they can provide useful information as well. PZT sensors can supplement other type of sensors designed for leakage detection, e.g., based on vapors or fluid detection out of the tank. In addition, emission of acoustic events during fluid pumping may provide useful information about pumps or valves condition.

In summary, all three types of PZT sensors showed potential for detecting acoustic emission events during the pressure test. However, their sensitivity and response characteristics varied, with some sensors being more sensitive to certain types of events. In the case of SMD07H and SMD07B sensors, a significant number of low-amplitude signals from water pumping were also recorded, which are not detectable by other type of sensors. The SMD05T04R111WL sensors did not obtain any leakage indications, which proves their limited effectiveness in the field of low frequency operation. The distribution of leakage signals for the other two types of sensors depends on the cross section of the tank. However, when analyzing all six sensors, no clear relationship between sensor sensitivity and the tank section in which they were installed was observed.

For comparative purposes, the response parameters of the sensor types were determined for the two selected events which were recorded by all sensors. The results are presented in Figure 31.

A direct comparison of response parameters for the same events related to structural damage reveals that the Steminc SMD07T05R411WL sensors record the highest amplitudes and signal energies. They also exhibit a significantly higher number of threshold exceedances, and thus also a longer duration of the signal. The frequency and rise time of the signals remain relatively similar for all sensors. Among the other two types of sensors, the differences are mainly in the signal energy, which is again related to the count of impulses with an amplitude higher than the threshold.

Thus, the comparison of determined response parameters confirms the earlier conclusions regarding the highest effectiveness of monitoring the composite tank structure using SMD07T05R411WL-type sensors: they not only generate the strongest signals in response to damage progression but also uniquely enable the effective detection of slow-varying effects, such as those caused by fluid pumping or leakage.

## 4. Summary

This paper presents a study of the application of PZT sensors for early detection of damage in chemical storage tanks. A complex scenario for guided wave propagation was considered as the monitored structure was composed of GFRP and PE layers. PZT sensors were installed on the GFRP layer and two types of damage—simulated cracks and chemical erosion—were introduced to the PE layer. In this study, it was possible to detect simulated erosion in the thermal weld of the PE layer with the diameter exceeding 8 mm as well as simulated cracks which exceeded 50% of the PE layer’s thickness. In addition, the usability of PZT sensor application as receivers of AE signals was verified. It was confirmed that AE events excited due to GFRP structure degradation during a pressure test can be successfully detected by PZT sensors. Those capabilities could be valuable for chemical storage tanks’ structural health monitoring and detection of events such as impacts or damage progression.

In this paper, a set of simple DIs which are able to capture some expected effects of elastic wave interaction with introduced damage was investigated. For further development, it is important to explore other signal characteristics or signal transformations, e.g., those presented in [70], which could be better tailored to the specific effects of elastic waves’ interactions with damage that occurs in such as case, in particular based on a deep understanding of interaction phenomena with use of numerical simulations or laser vibrometer measurements. Also, the presented results with respect to the efficiency of selected DIs were obtained under laboratory conditions. It was demonstrated that various operational and environmental conditions may impact guided-wave-based structural health monitoring [71]; in particular, the temperature, pressure, moisture, vibration or sensor’s bonding deterioration can affect signals acquired by PZT sensors. In [72], it was shown that temperature rise by about 30 °C can affect corr and divAmp DIs values in a similar way as the damage introduced in this study. In the case of chemical storage tanks, it can be expected that the temperature and internal pressure variability can be significantly higher during their operation compared to laboratory conditions. Therefore, it is important to estimate their influence on signals and their characteristics in order to determine the calibration tolerance of SHM system based on the application of PZT sensors in a real case. Nevertheless, many efficient algorithms for DI compensation were designed based on proper signal transformations or with the use of properly defined baseline signals database [72,73,74,75,76]. Additionally, it can be expected that the application of novel machine learning algorithms to signal classification under varying measurement conditions will lead to further breakthroughs in that area [77]. In this paper, it has been demonstrated that based on signal characteristics sensitive to the expected effects of elastic wave interaction with damage, e.g., signal amplitude and phase changes, it is possible to capture damage scenarios which can occur during the operation of chemical storage tanks; therefore, PZT sensors can be considered as suitable for SHM system development in that case.

In summary, PZT sensors proved their potential as effective tools for monitoring the structural integrity of chemical storage tanks, but practical implementation may require addressing various operational and environmental challenges. Further research and development are needed to optimize PZT sensor systems for specific applications and conditions.

## Data Availability

Data used in this study are available on-demand from the corresponding author.

## Abbreviations

The following abbreviations are used in this manuscript:

AE	Acoustic Emission
DI (DIs)	Damage Index (Damage Indices)
GFRP(s)	Glass Fiber Reinforced Polymer(s)
PE	polyethylene
PZT	lead zirconate titanate
SHM	Structural Health Monitoring
